# Supplementary figures and images for: The Hippo kinase cascade regulates a contractile cell behavior and cell density in a close unicellular relative of animals
Source: bioRxiv. 2024 Jan 16:2023.07.25.550562. Originally published 2023 Jul 25. Preprint. [Version 2] doi: 10.1101/2023.07.25.550562 (PMC10402117; doi:10.1101/2023.07.25.550562)

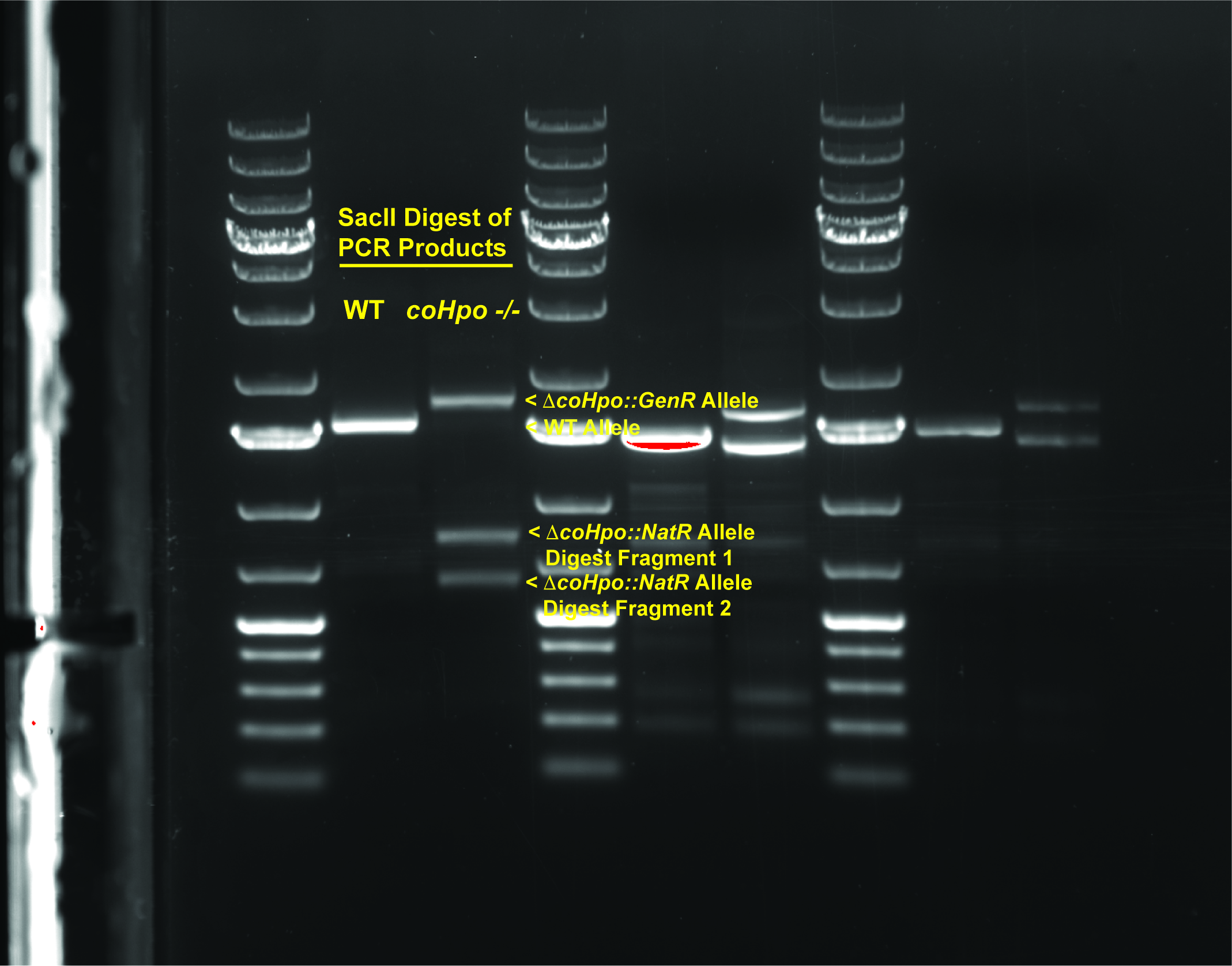

Supplement: Supplement 6 [file media-6.zip › source data/Figure 1-supplement 1-source data 2.tif]

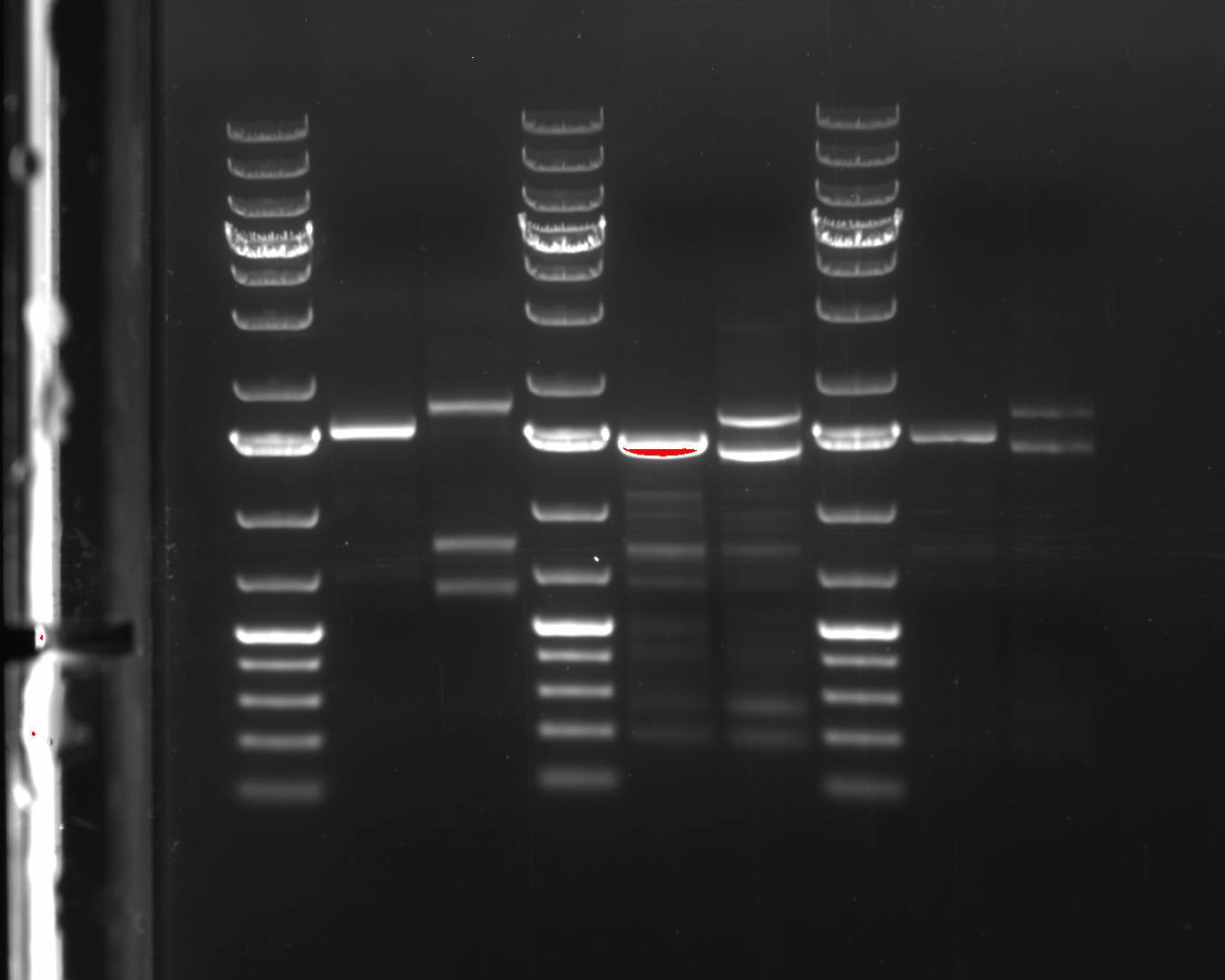

Supplement: Supplement 6 [file media-6.zip › source data/Figure 1-supplement 1-source data 3.tif]

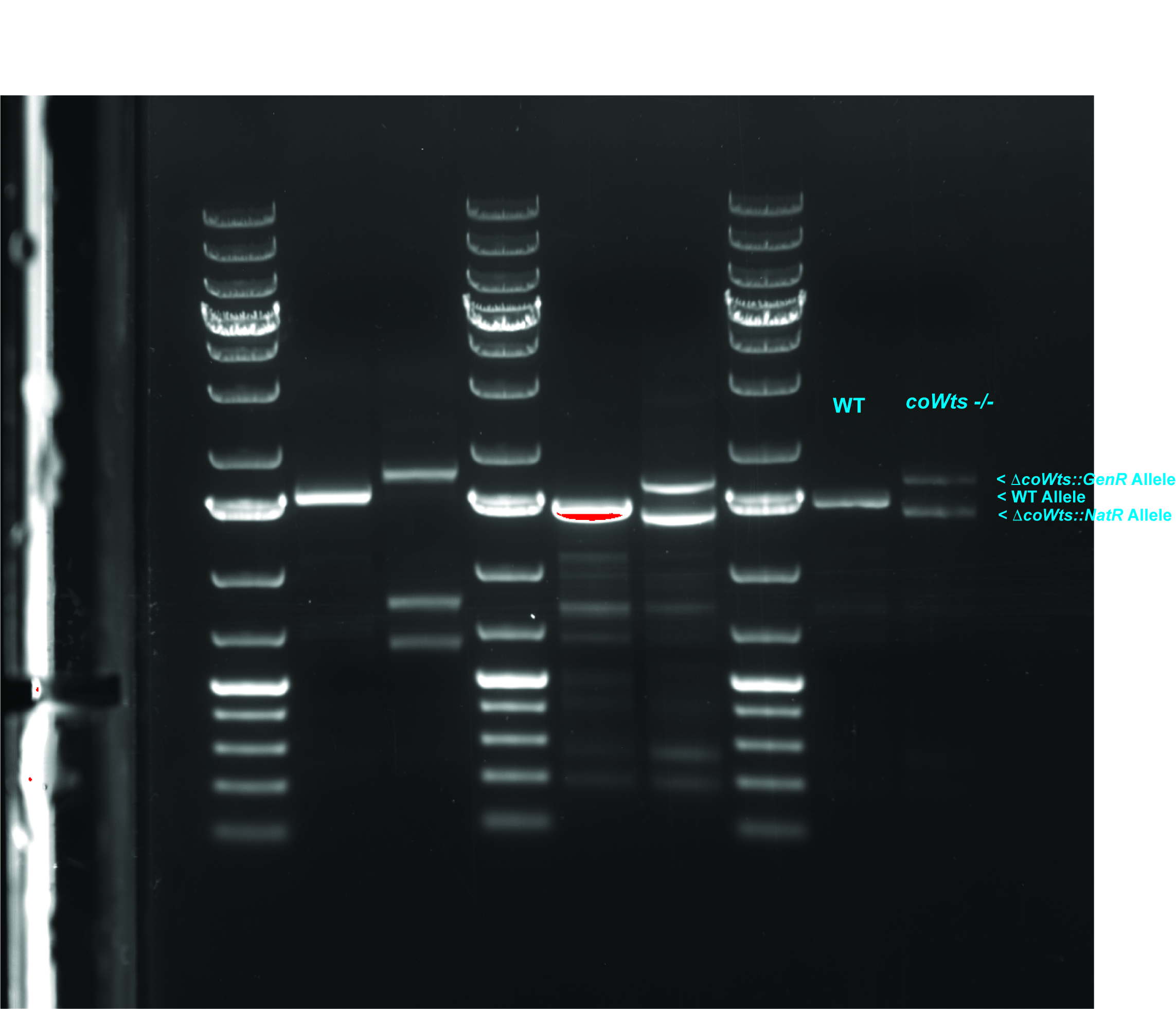

Supplement: Supplement 6 [file media-6.zip › source data/Figure 1-supplement 1-source data 4.tif]
