# Supplementary material for: The Hippo kinase cascade regulates a contractile cell behavior and cell density in a close unicellular relative of animals
Source: bioRxiv. 2024 Jan 16:2023.07.25.550562. Originally published 2023 Jul 25. Preprint. [Version 2] doi: 10.1101/2023.07.25.550562 (PMC10402117; doi:10.1101/2023.07.25.550562)
Supplement: Supplement 10 [file NIHPP2023.07.25.550562v2-supplement-10.pdf]

bioRxiv preprint doi: https://doi.org/10.1101/2023.07.25.550562; this version posted January 16, 2024. The copyright holder for this preprint (which was not certified by peer review) is the author/funder, who has granted bioRxiv a license to display the preprint in perpetuity. It is made available under aCC-BY 4.0 International license.

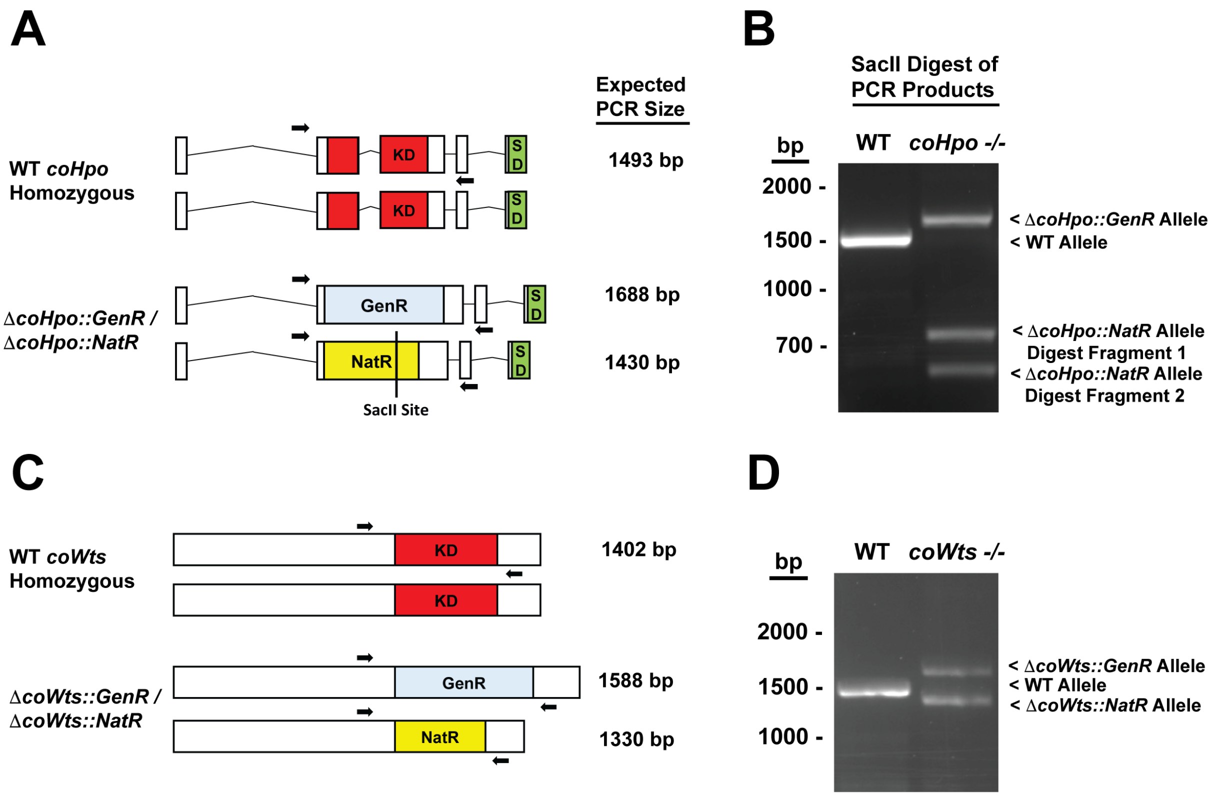

Figure 1- figure supplement 1. Knockout of *coHpo and coWts*. (A) A diagram of the *coHpo* genomic loci in WT or a putative *coHpo -/-* knockout line is shown. "KD" indicates kinase domain, "SD" indicates SARAH domain, "GenR" indicates a geneticin resistance cassette, and "NatR" indicates a nourseothricin resistance cassette. Arrows indicate a pair of primers used in PCR reactions. The same primer pair were used in all PCRs shown. Expected PCR product sizes for different alleles are given. A SacII site present only within the allele containing the NatR cassette is shown. **(B)** PCR products from WT and *coHpo -/-* cells using primers shown in (A). Because the product sizes for the WT and *ΔcoHpo::NatR* alleles are close in size, we digested PCR products with SacII, which uniquely cuts the *ΔcoHpo::NatR* allele. The *coHpo -/-* cell line shows the expected products for a homozygous knockout. Sequencing of PCR products confirmed the identity of the WT, *ΔcoHpo::GenR*, and *ΔcoHpo::NatR* alleles. **(C)** A diagram of the *coWts* genomic loci in WT or a putative *coWts -/-* knockout line is shown. "KD" indicates kinase domain, "GenR" indicates a geneticin resistance cassette, and "NatR" indicates a nourseothricin resistance cassette. Arrows indicate a pair of primers used in PCR reactions. The same primer pair were used in all PCRs shown. **(D)** PCR products from WT and *coHpo -/-* cells using primers shown in (C). The *coWts -/-* cell line shows the expected products for a homozygous knockout.

bioRxiv preprint doi: https://doi.org/10.1101/2023.07.25.550562; this version posted January 16, 2024. The copyright holder for this preprint (which was not certified by peer review) is the author/funder, who has granted bioRxiv a license to display the preprint in perpetuity. It is made available under aCC-BY 4.0 International license.

Sequencing of PCR products confirmed the identity of the WT, *ΔcoWts::GenR*, and *ΔcoWts::NatR* alleles.

bioRxiv preprint doi: https://doi.org/10.1101/2023.07.25.550562; this version posted January 16, 2024. The copyright holder for this preprint (which was not certified by peer review) is the author/funder, who has granted bioRxiv a license to display the preprint in perpetuity. It is made available under aCC-BY 4.0 International license.

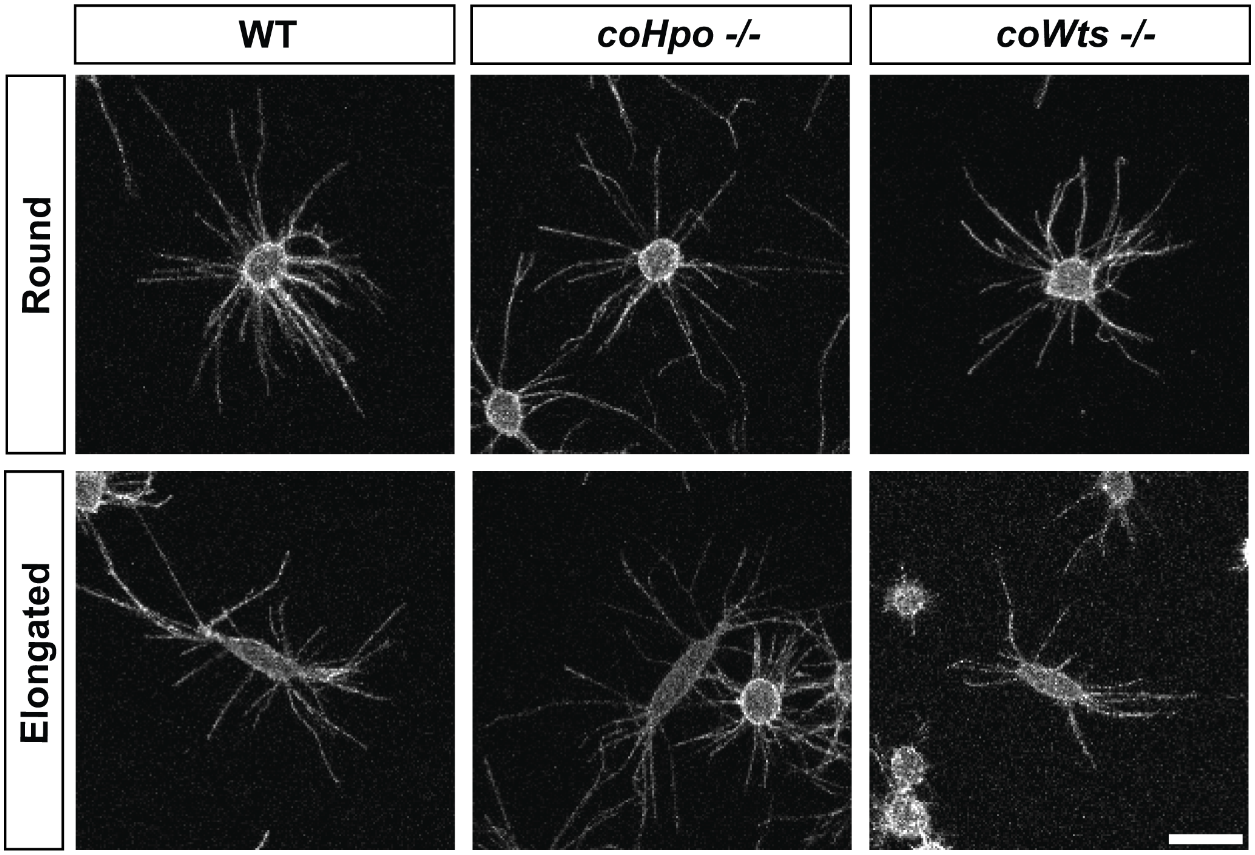

Figure 3- figure supplement 1. Filopodia are enriched at the poles of elongated cells. Adherent cells stably expressing NMM-Venus, a plasma membrane marker which allows for visualization of filopodia, were imaged live by confocal microscopy. Scale bar is 10 microns.

bioRxiv preprint doi: https://doi.org/10.1101/2023.07.25.550562; this version posted January 16, 2024. The copyright holder for this preprint (which was not certified by peer review) is the author/funder, who has granted bioRxiv a license to display the preprint in perpetuity. It is made available under aCC-BY 4.0 International license.

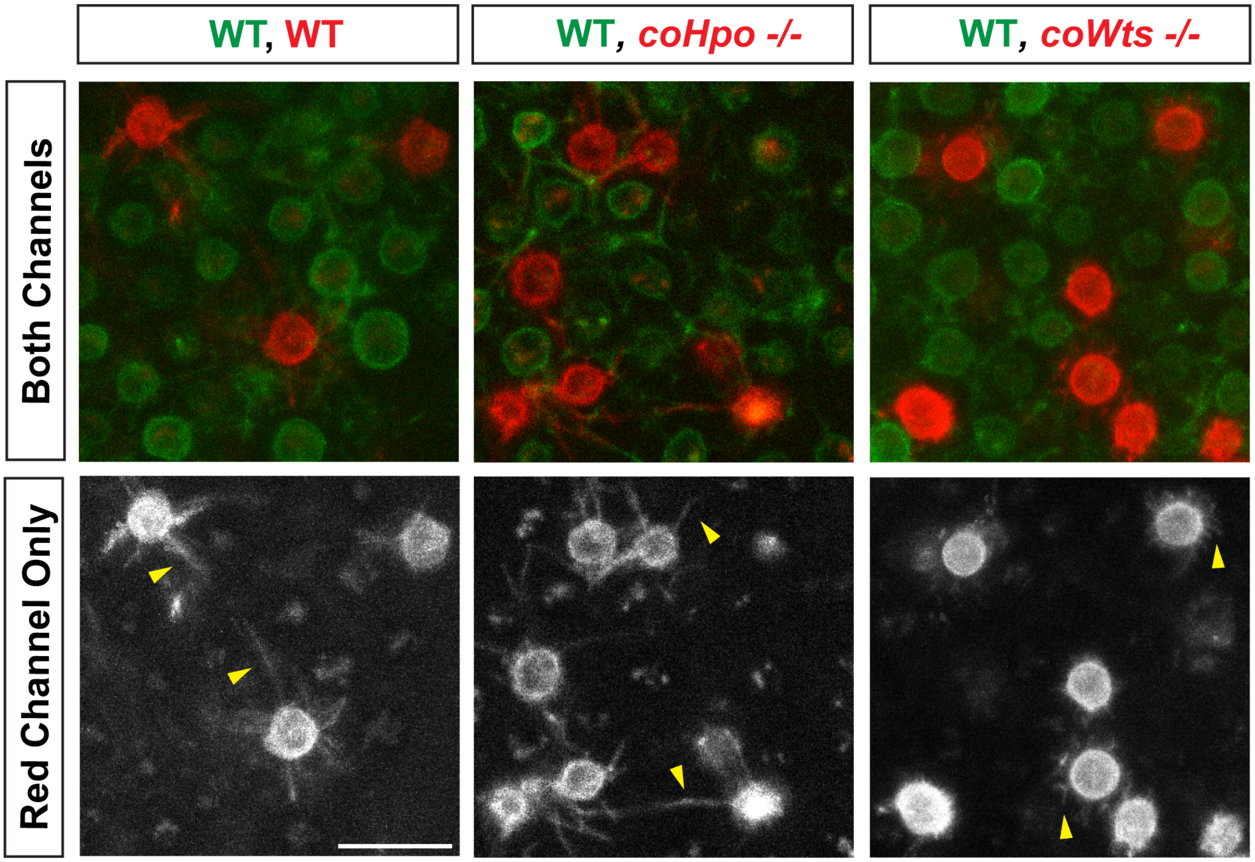

Figure 5- figure supplement 1. *coWts* -/- cells within multicellular aggregates show altered filopodial morphology and reduced filopodial length. WT cells stably expressing NMM-Venus, which allows visualization of filopodia, were mixed with WT, *coHpo* -/-, or *coWts* -/- cells stably expressing NMM-mScarlet at a 9:1 cell ratio and were inoculated into low-adherence wells to induce aggregate formation. Cells within aggregates were then imaged three days after aggregate induction. Yellow arrowheads indicate examples of filopodia. Scale bar is 10 microns.

bioRxiv preprint doi: https://doi.org/10.1101/2023.07.25.550562; this version posted January 16, 2024. The copyright holder for this preprint (which was not certified by peer review) is the author/funder, who has granted bioRxiv a license to display the preprint in perpetuity. It is made available under aCC-BY 4.0 International license.

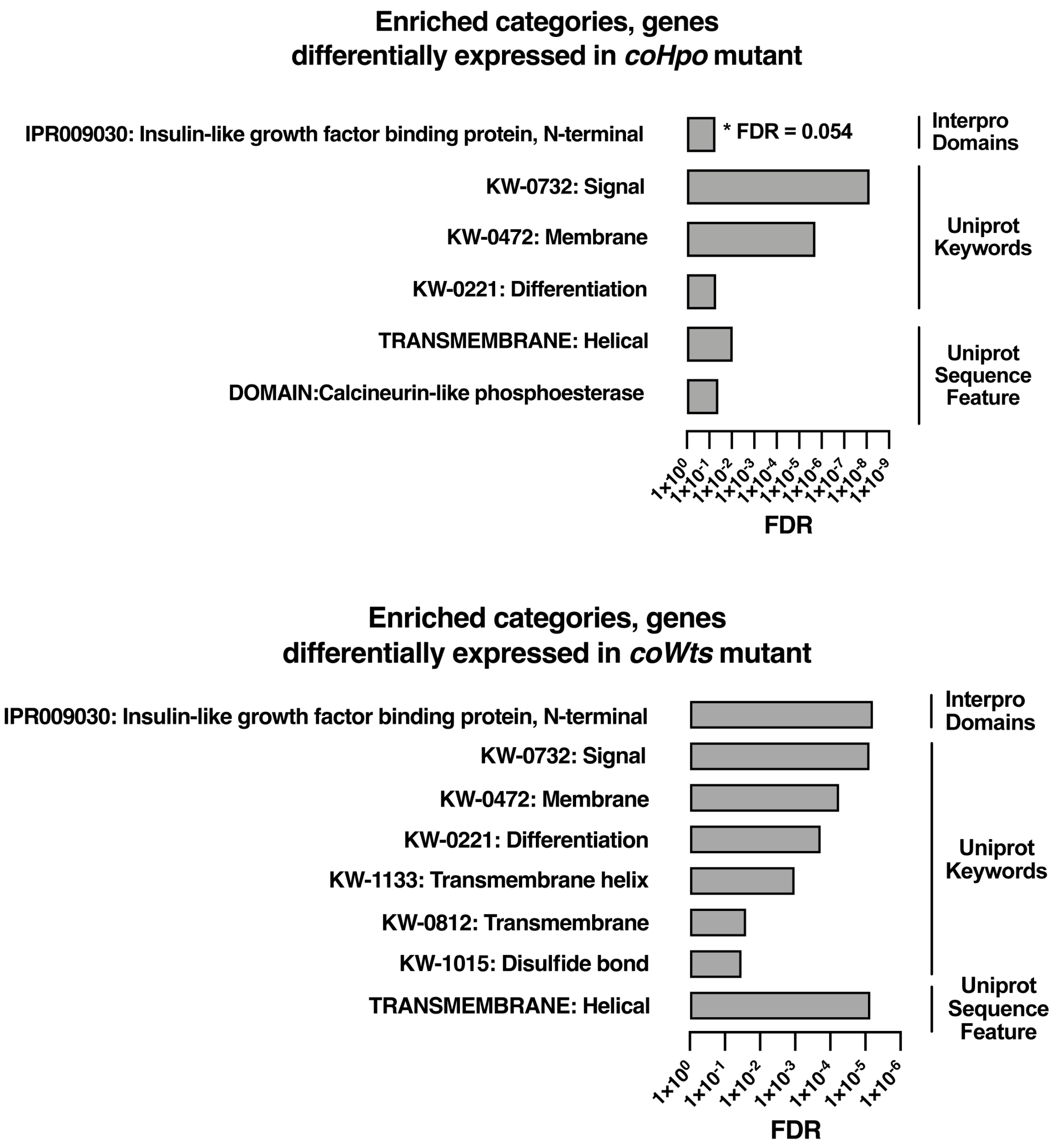

Figure 8- figure supplement 1. Enrichment analysis for genes differentially expressed in coHpo or coWts mutant cells. Categories with FDR < 0.05 are shown, except where FDR is indicated. Interpro or Uniprot accession numbers are given for enriched categories where appropriate. FDR: false discovery rate.

bioRxiv preprint doi: https://doi.org/10.1101/2023.07.25.550562; this version posted January 16, 2024. The copyright holder for this preprint (which was not certified by peer review) is the author/funder, who has granted bioRxiv a license to display the preprint in perpetuity. It is made available under aCC-BY 4.0 International license.

Video S1. Time-lapse microscopy of adherent WT *Capsaspora* cells. This video serves as a control for Videos S2 and S3.

Video S2. Time-lapse microscopy of adherent *coHpo -/-* cells.

Video S3. Time-lapse microscopy of adherent *coWts -/-* cells.

Video S4. Time-lapse confocal microscopy of adherent WT cells expressing Lifeact-mScarlet. This video serves as a control for Video S5.

Video S5. Time-lapse confocal microscopy of adherent *coHpo -/-* cells expressing Lifeact-mScarlet.

bioRxiv preprint doi: https://doi.org/10.1101/2023.07.25.550562; this version posted January 16, 2024. The copyright holder for this preprint (which was not certified by peer review) is the author/funder, who has granted bioRxiv a license to display the preprint in perpetuity. It is made available under aCC-BY 4.0 International license.

Supplementary File 1. Genes with actin binding functional annotation showing differential expression in *coYki* mutant cells and also exhibiting differential expression in *coHpo* and/or *coWts* mutant cells

Supplementary File 2. Lists of genes differentially expressed in *coHpo* mutant cells, coWts mutant cells, and the overlapping set of genes differentially expressed in *coHpo*, *coWts*, and *coYki* mutant cells.

Supplementary File 3. Sequences of synthesized gene fragments used in this study

bioRxiv preprint doi: https://doi.org/10.1101/2023.07.25.550562; this version posted January 16, 2024. The copyright holder for this preprint (which was not certified by peer review) is the author/funder, who has granted bioRxiv a license to display the preprint in perpetuity. It is made available under aCC-BY 4.0 International license.

Figure 1-supplement 2-source data 1. Unedited gel image used to generate panel B.

Figure 1-supplement 2-source data 2. Uncropped annotated gel image used to generate panel B.

Figure 1-supplement 2-source data 3. Unedited gel image used to generate panel D.

Figure 1-supplement 2-source data 4. Uncropped annotated gel image used to generate panel D.
