# Supplementary material for: The Hippo kinase cascade regulates a contractile cell behavior and cell density in a close unicellular relative of animals
Source: bioRxiv. 2023 Jul 25:2023.07.25.550562. Preprint. [Version 1] doi: 10.1101/2023.07.25.550562 (PMC10402117; doi:10.1101/2023.07.25.550562)
Supplement: Supplementary file 1 [file NIHPP2023.07.25.550562v1-supplement-8.pdf]

bioRxiv preprint doi: https://doi.org/10.1101/2023.07.25.550562; this version posted July 25, 2023. The copyright holder for this preprint (which was not certified by peer review) is the author/funder, who has granted bioRxiv a license to display the preprint in perpetuity. It is made available under aCC-BY 4.0 International license.

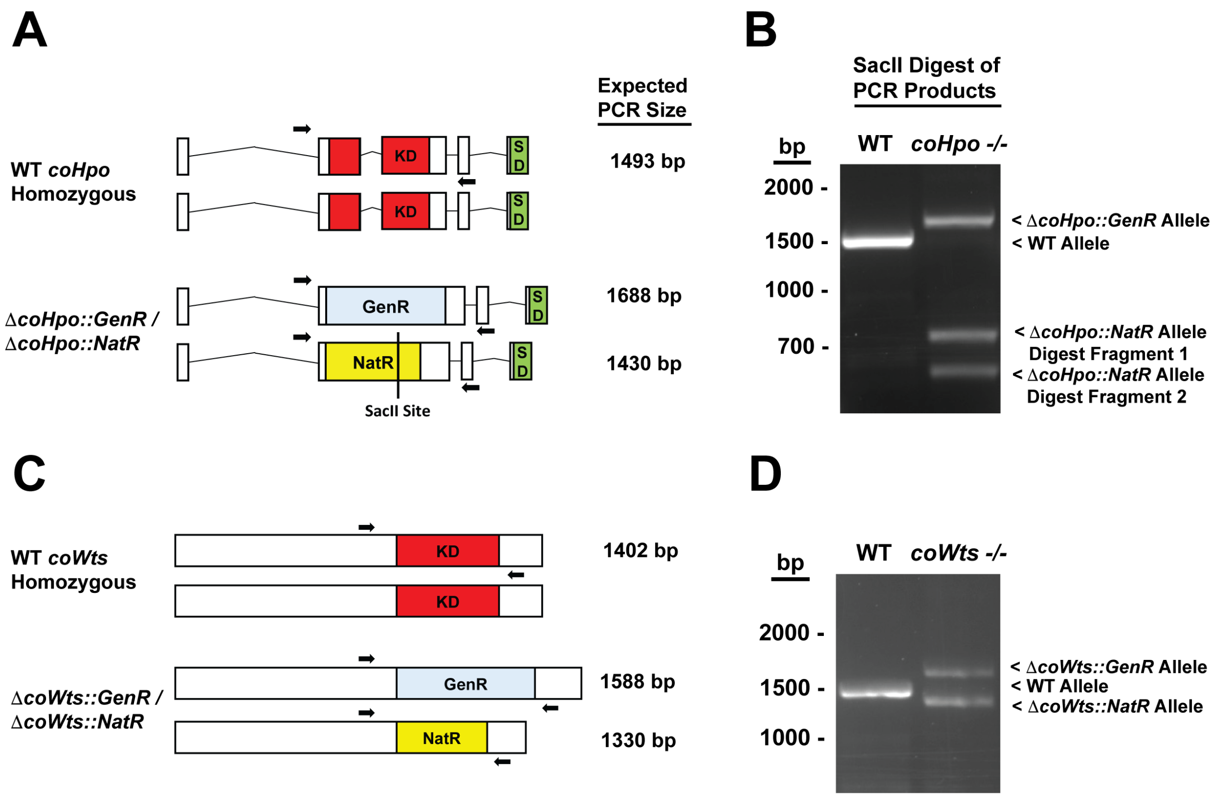

Figure 1- figure supplement 1. Knockout of *coHpo and coWts*. (A) A diagram of the *coHpo* genomic loci in WT or a putative *coHpo -/-* knockout line is shown. "KD" indicates kinase domain, "SD" indicates SARAH domain, "GenR" indicates a geneticin resistance cassette, and "NatR" indicates a nourseothricin resistance cassette. Arrows indicate a pair of primers used in PCR reactions. The same primer pair were used in all PCRs shown. Expected PCR product sizes for different alleles are given. A SacII site present only within the allele containing the NatR cassette is shown. **(B)** PCR products from WT and *coHpo -/-* cells using primers shown in (A). Because the product sizes for the WT and *ΔcoHpo::NatR* alleles are close in size, we digested PCR products with SacII, which uniquely cuts the *ΔcoHpo::NatR* allele. The *coHpo -/-* cell line shows the expected products for a homozygous knockout. Sequencing of PCR products confirmed the identity of the WT, *ΔcoHpo::GenR*, and *ΔcoHpo::NatR* alleles. **(C)** A diagram of the *coWts* genomic loci in WT or a putative *coWts -/-* knockout line is shown. "KD" indicates kinase domain, "GenR" indicates a geneticin resistance cassette, and "NatR" indicates a nourseothricin resistance cassette. Arrows indicate a pair of primers used in PCR reactions. The same primer pair were used in all PCRs shown. **(D)** PCR products from WT and *coHpo -/-* cells using primers

bioRxiv preprint doi: https://doi.org/10.1101/2023.07.25.550562; this version posted July 25, 2023. The copyright holder for this preprint (which was not certified by peer review) is the author/funder, who has granted bioRxiv a license to display the preprint in perpetuity. It is made available under aCC-BY 4.0 International license.

shown in (C). The *coWts -/-* cell line shows the expected products for a homozygous knockout. Sequencing of PCR products confirmed the identity of the WT, *ΔcoWts::GenR*, and *ΔcoWts::NatR* alleles.

bioRxiv preprint doi: https://doi.org/10.1101/2023.07.25.550562; this version posted July 25, 2023. The copyright holder for this preprint (which was not certified by peer review) is the author/funder, who has granted bioRxiv a license to display the preprint in perpetuity. It is made available under aCC-BY 4.0 International license.

Video S1. Time-lapse microscopy of adherent WT *Capsaspora* cells. This video serves as a control for Videos S2 and S3.

Video S2. Time-lapse microscopy of adherent *coHpo -/-* cells.

Video S3. Time-lapse microscopy of adherent *coWts -/-* cells.

Video S4. Time-lapse confocal microscopy of adherent WT cells expressing Lifeact-mScarlet. This video serves as a control for Video S5.

Video S5. Time-lapse confocal microscopy of adherent *coHpo -/-* cells expressing Lifeact-mScarlet.

bioRxiv preprint doi: https://doi.org/10.1101/2023.07.25.550562; this version posted July 25, 2023. The copyright holder for this preprint (which was not certified by peer review) is the author/funder, who has granted bioRxiv a license to display the preprint in perpetuity. It is made available under aCC-BY 4.0 International license.

Data S1. Sequences of synthesized gene fragments used in this study

bioRxiv preprint doi: https://doi.org/10.1101/2023.07.25.550562; this version posted July 25, 2023. The copyright holder for this preprint (which was not certified by peer review) is the author/funder, who has granted bioRxiv a license to display the preprint in perpetuity. It is made available under aCC-BY 4.0 International license.

Figure 1-supplement 2-source data 1. Unedited gel image used to generate panel B.

Figure 1-supplement 2-source data 2. Uncropped annotated gel image used to generate panel B.

Figure 1-supplement 2-source data 3. Unedited gel image used to generate panel D.

Figure 1-supplement 2-source data 4. Uncropped annotated gel image used to generate panel D.
